# Mechanical Properties of Cement Reinforced with Pristine and Functionalized Carbon Nanotubes: Simulation Studies

**DOI:** 10.3390/ma15217734

**Published:** 2022-11-03

**Authors:** Rosario G. Merodio-Perea, Isabel Lado-Touriño, Alicia Páez-Pavón, Carlos Talayero, Andrea Galán-Salazar, Omar Aït-Salem

**Affiliations:** Department of Industrial and Aerospace Engineering, School of Architecture, Engineering and Design, Universidad Europea de Madrid, 28670 Villaviciosa de Odón, Spain

**Keywords:** cement, carbon nanotubes, molecular dynamics, mechanical properties, pull-out, interfacial shear strength

## Abstract

Concrete is well known for its compression resistance, making it suitable for any kind of construction. Several research studies show that the addition of carbon nanostructures to concrete allows for construction materials with both a higher resistance and durability, while having less porosity. Among the mentioned nanostructures are carbon nanotubes (CNTs), which consist of long cylindrical molecules with a nanoscale diameter. In this work, molecular dynamics (MD) simulations have been carried out, to study the effect of pristine or carboxyl functionalized CNTs inserted into a tobermorite crystal on the mechanical properties (elastic modulus and interfacial shear strength) of the resulting composites. The results show that the addition of the nanostructure to the tobermorite crystal increases the elastic modulus and the interfacial shear strength, observing a positive relation between the mechanical properties and the atomic interactions established between the tobermorite crystal and the CNT surface. In addition, functionalized CNTs present enhanced mechanical properties.

## 1. Introduction

Recent years have witnessed an increasing interest in cement composites with the incorporation of different types of carbon nanotubes (CNTs) [1,2,3,4]. These are long cylindrical carbon molecules found by Iijima in 1995 [5]. They look like a layer of graphene rolled up on itself. Different authors [6,7] highlighted their excellent electrical, chemical and mechanical properties, which have revolutionized composite materials, microelectronics, biomedical applications and energy storage [8,9,10]. CNTs are characterized by a high elastic modulus as well as tensile strength [11], which makes them a very suitable option to reinforce materials such as cement. Therefore, it is possible to obtain composite materials with improvement in both tensile and compression strengths, as well as better durability, since crack propagation is inhibited [12,13,14,15,16,17].

Although most studies on cement reinforced with CNTs have been carried out only on a laboratory scale, some works attempted to extend the use of these materials to the large scale. The most critical point of the problem is the production of CNTs with controllable size and length. Due to great advances in material science, CNTs have already been mass produced in orders of several to several tens of kilograms per hour [18,19]. Large-scale production of CNT-cement composites has also been reported by several authors. Silva et al. [20] patented a method to produce CNTs embedded in a cement matrix in a continuous and large-scale stage. The authors state that this process could produce several tons per day and thus, be appropriate for the conventional cement industry. Jianguo et al. [21] developed a method to disperse CNTs in a cement matrix, appropriate for large-scale application. Jianlin et al. [22] described the fabrication of an intelligent concrete reinforced with CNTs and graphene oxide, suitable for production in industrial plants. As a conclusion, it can be said that the fabrication of CNT-cement composites is not yet completely implemented on an industrial scale. However, an increasing number of studies describing the mass production process of these materials is observed.

Despite the huge interest in CNTs as a reinforcement, there are certain problems to disperse them homogeneously in the cement matrix. This remains an obstacle to design new materials with enhanced properties. CNTs tend to agglomerate and form bundles due to Van der Waals interactions. Hence, dispersion in the cement matrix seems difficult and strong adhesion is not favored. Different methods have been tested to improve the dispersion of CNTs in the cement matrix. Some of these methods are based on ultrasonication combined with a functionalizing agent, which modifies the CNT surface to ensure a better dispersion [23]. Amid these methods, the addition of carboxyl groups to the CNT structure must be mentioned, since it boosts hydrophilic features and chemical bonding between CNTs and cement, causing improved adhesion between components [24,25].

Properties of both pure and carbon-reinforced cement have been studied by different authors in the literature using molecular dynamics techniques (MD). Murray et al. [26] simulated tensile and compression behavior of hydrated calcium silicate (C-S-H), which is the main component of Portland cement. Fu et al. [27] evaluated the mechanical properties of hydrated calcium silicate structures based on tobermorite 11 Å, through tensile-compression cycles with LAMMPS software. In addition, Huang et al. [28] analyzed the influence of the Ca/Si ratio and temperature on the uniaxial mechanical properties of C-S-H gel.

The mechanical properties of cement reinforced with carbon nanostructures were calculated by several authors. Sánchez et al. [29] and Hou et al. [30] studied the influence of functionalization on the interaction between graphene oxide and tobermorite through MD. In both cases, the authors observed an increase in the adhesion energy when functionalizing the graphene oxide surface with different functional groups. The effect of quirality and the CNT diameter on the mechanical properties of the tobermorite 11 Å was analyzed by Lushnikova et al. [31], as well as the mechanical properties for plain and CNT reinforced cement [32]. The enhancement of mechanical properties of reinforced cement with carbon nanotubes is also found in different papers by other authors [33,34,35,36].

Experimental measurement of the interfacial shear strength (ISS) in a composite can be carried out through methods, such as macromechanical, fragmentation, pull-out, microdroplet, push-out and push-in tests [37,38,39,40,41,42]. These methods encounter several technical difficulties and, to the best of our knowledge, they have not been applied to the study of CNTs-cement interfaces. Another way to characterize these interfaces is to calculate the ISS through MD techniques. There are several such studies applied to composite materials with a polymeric matrix [43,44]. However, ISS simulations are scarce for nanocarbon-cement compounds and most of them have been applied to graphene and its derivatives [45,46].

In our previous research, the interaction between tobermorite and different CNTs was studied through experimental techniques, MD and the finite element method [47,48], obtaining quite promising results. These studies show the relation between the functionalization of the CNT surface, concentration and the type of CNT (single- or multi-wall) and the mechanical properties of the material; and agree with those previously found by Zhao et al. [49].

The present work presents a study of the mechanical properties of a tobermorite 11 Å crystal (elastic modulus E and ISS), with pristine CNT or functionalized CNTs with varying numbers of carboxyl groups. The MD results show that the incorporation of CNTs enhances the mechanical properties of the tobermorite and the improvement relates to the degree of functionalization of the CNT surface. The larger the number of carboxyl groups on the CNT surface, the higher the values obtained for E and ISS. These results are a consequence of better interactions that are established at the cement–CNT interface. 

## 2. Materials and Methods

### 2.1. Model Systems

**Figure 1:**
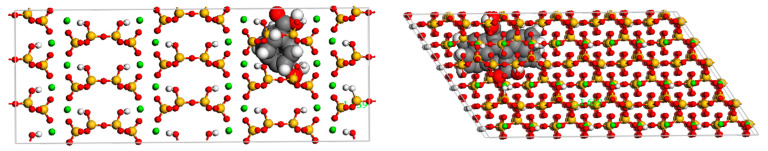
Side (**left**) and top (**right**) view of a SWCNT (2,2), which is functionalized with two COOH groups, embedded in a tobermorite crystal 11 Å. Red: oxygen, Yellow: silicon, Green: calcium, White: hydrogen, Grey: carbon.

**Figure 2:**
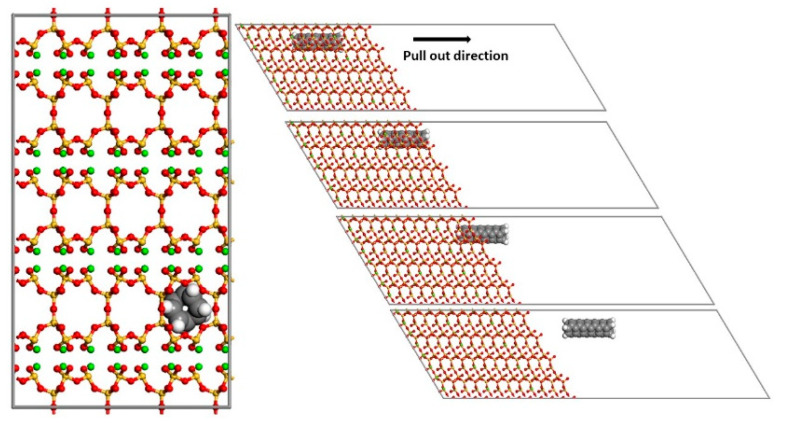
Side (**left**) and top (**right**) view of the models used for calculating ISS, showing an embedded pristine SWCNT (2,2) in a tobermorite matrix. The picture on the right depicts the pulling out process. Red: oxygen, Yellow: silicon, Green: calcium, White: hydrogen, Grey: carbon.

With the aim of analyzing the effect of the addition of pristine and functionalized single-walled CNTs (SWCNTs) on the mechanical properties of tobermorite 11 Å, different crystalline models were used. A SWCNT (2,2) was inserted in one of the interstices of the tobermorite crystal, as shown in Figure 1 and Figure 2. It was decided to work with a SWCNT (2,2) due to its small diameter (2.71 Å), which does not cause much distortion on the tobermorite structure. 

When calculating the ISS, the pulling out of the CNT must be done in a certain direction, pulling it out from the crystalline structure until it does not interact with it anymore. Therefore, the length of the cell has been increased to 500 Å (see Figure 2) in the pulling-out direction in order to simulate the pull-out process and avoid the interaction with the surrounding cells and the crystal. 

**Figure 3:**
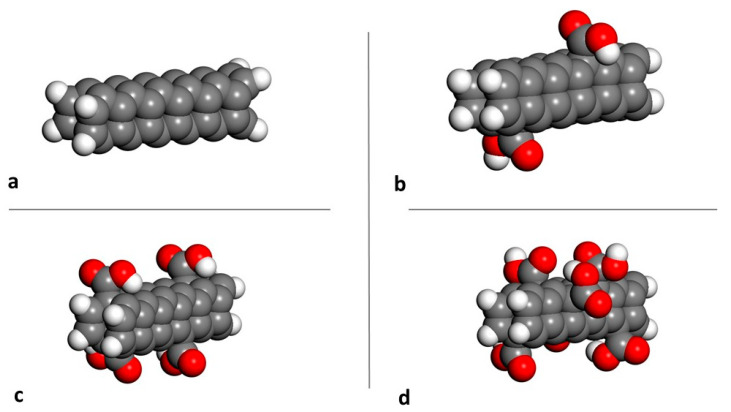
(**a**) pristine SWCNT (2,2) and functionalized with (**b**) 2 COOH groups, (**c**) 4 groups and (**d**) 6 groups. Red: oxygen, White: hydrogen, Grey: carbon.

Figure 3 depicts the different pristine and functionalized CNTs that were used to calculate the mechanical properties.

Geometrical features of the models used to calculate the mechanical properties are listed in Table 1.

### 2.2. Calculation Method

The Forcite Module of Materials Studio Software [50] was used to calculate mechanical properties. Within the Forcite Module, the NPT ensemble was selected (N: number of particles, P: pressure, T: temperature), with both a constant temperature (298 K with a Nose-Hoover thermostat [51]) and pressure (1 × 10^−4^ GPa with a Berendsen barostat [52]). The MD simulation was carried out for 6000 ps simulation time and 1 fs as a time interval. The chosen times were enough to achieve the equilibrium in the potential energy of the system.

The forcefield used to calculate the interaction between the cement and the CNT was the condensed-phase optimized molecular potential for atomistic simulation studies forcefield (COMPASSII) [53]; a forcefield based on ab initio calculations that allows describing the structure and properties of molecules and condensed phase systems in a wide range of temperature and pressure values. COMPASSII has been successfully applied in the simulation of systems containing CNTs and different materials derived from cement [37,54,55,56,57,58,59].

To calculate the mechanical properties (E, ν, G and K), the elastic method was used, in which the response to an applied strain is derived from the second derivative of the potential energy with respect to strain. The relaxation of the system under applied strain is determined from the Hessian matrix. This method was applied to the last 10 frames of the MD trajectory and the elastic constants, K and G, were averaged over all frames. The mentioned method has been used by other authors, who obtained results comparable to the experimental values of the mechanical properties of both the C-S-H gel and polymers reinforced with CNTs [54,57,59]. Using the Voigt-Reuss-Hill (VRH) approximation [60], it is possible to compute E and ν, with the following equations, respectively:

(1) $$E=\frac{9\cdot K\cdot G}{3\cdot K+G}$$

(2) $$\nu =\frac{3\cdot K-2\cdot G}{2\cdot \left(3\cdot K+G\right)}$$

The Gou et al. [61] equation was used in order to calculate the ISS. They defined the pullout energy, E_pullout_, as the energy difference between the fully embedded CNT configuration and the complete pullout configuration. In turn, E_pullout_ can be related to the ISS through the following equation:

(3) $$\tau i=\frac{E_{pullout}}{\pi \cdot r\cdot L^{2}}$$

where r and L are the radius and length of the CNT, respectively.

## 3. Results and Discussion

### 3.1. Mechanical Properties

Table 2 shows the values obtained for bulk modulus (K), shear modulus (G), Young’s modulus (E) and Poisson’s ratio (ν), which were calculated using Equations (1) and (2).

Several authors have calculated tobermorite modules by MD, obtaining similar results. B. Bhuvaneshwari [55] computed all the modules for the three types of tobermorite (9 Å, 11 Å and 14 Å) and jennite, obtaining very similar values to those presented in this work. M. Arar [62] also reported similar results.

On the other hand, other authors have studied the mechanical behavior of tobermorite when adding pristine [63,64] and functionalized [65,66,67,68] CNTs, showing that the tensile strength of C-S-H reinforced with CNT (functionalized and non-functionalized) is significantly enhanced in the CNT direction, when compared to pure C-S-H.

**Figure 4:**
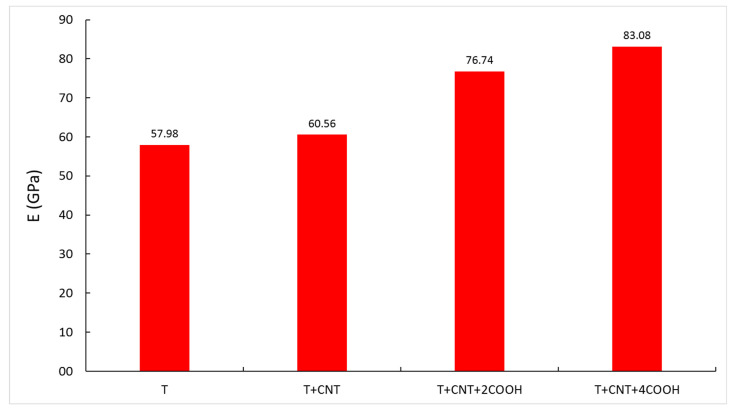
Young’s modulus for all models.

To better understand the results:

Figure 4 represents the E obtained for each structure. It can be clearly seen that E increases when adding pristine and functionalized SWCNTs. In addition, functionalized CNTs perform better. The enhancement seems to be a consequence of the interaction between the tobermorite and the functionalized CNT thanks to the added functional groups. The larger the number of carboxyl groups, the higher the elastic modulus.

It is important to mention that the experimental values seem to be lower than the computed values. Velez et al. [69] measured the elastic modulus of different clinkers contained in the cement as a function of porosity and found out higher values for those materials with null porosity. Jennings [70] proposed a colloidal model for the cement structure, which consists of globular C-S-H particles. Depending on the compaction level of the cement, it is possible to obtain two different structures: high-density C-S-H (HD C-S-H) and low-density C-S-H (LD C-S-H), with a greater average porosity for the second one. There exists a simple method to compute E as a function of porosity using the equation proposed by Knudsen and Helmuth [71]:

(4) $$E=E_{o}\cdot e^{-3.4\cdot p}$$

where E_o_ is the elastic modulus in the absence of porosity, *p* is the porosity and 3.4 represents a coefficient obtained from many experimental measurements [69].

Table 3 shows the value of E as a function of the average porosity for HD (0.26) and LD (0.36) structures. As it is possible to appreciate, an increase in porosity causes a significant decrease in E. In addition, our values for tobermorite are similar to those measured by Arar [62], who obtained 15 GPa and 25 GPa for LD and HD, respectively. Moreover, González et al. [72] measured 32.2 GPa for HD and 16.3 GPa for LD, while Fu et al. [27] attained 31.45 GPa (HD) and 18.11 GPa (LD). The experimental values for HD and LD were measured by Constantinides [73] and Keinde [74] using nanoindentation techniques, who obtained 21.7 GPa for LD and 29 GPa for HD.

### 3.2. Interfacial Shear Strength (ISS)

Table 4 shows the values obtained for ISS and non-bond energy for pristine CNTs and CNTs functionalized with four and six COOH groups. Moreover, the ΔE_non-bond_, which represents the difference between the non-bond energies of the structures without and with the CNT inside the crystal (E_pulled-out configuration non-bond_ − E_fully embedded configuration non-bond_), is also shown in this Table. The structures with functionalized CNTs show higher ISS and ISS is increased for CNT with larger number of COOH groups. Since there is no chemical bonding at the interface between the CNT and the tobermorite, the interaction between both is mainly dominated by Van der Waals and electrostatic forces. When the CNT is removed from the structure, these interactions disappear and a decrease in the non-bonding energy is observed. The values of ΔE_non-bond_ are positive as E_non-bond_ is negative. CNTs with polar groups, such as COOH, show better values (more negative) of the E_non-bond_, because the interaction with the tobermorite structure is enhanced, which, in turn, increases ISS [37,75,76].

As is seen from Table 4, there is a positive relation between E_non-bond_ and ISS.

### 3.3. Hydrogen Bonds (H-Bonds)

CNTs functionalized with COOH groups can establish H bonds with oxygen atoms in the tobermorite. Geometry requisites that have been used to define the presence of a H bond are: the distance between H from the donor group (D) and O from the acceptor group (A) is lower than 2.5 Å and the angle DHA is higher than 90°. Several authors [29,37,43,64,77] have found a positive relation between the mechanical properties of a cement matrix reinforced with carbon nanostructures and interactions, chemical bonds or non-bond interactions, which are established at the interface of the materials. 

The number of H bonds (NHb) established between the CNT and the tobermorite (for the models used to compute E) and the average length of those bonds can be seen in Table 5. Bond lengths between the CNT and the matrix are slightly larger for the structures containing pristine CNTs.

The results for the models used for the pull-out process are listed in Table 6. 

**Figure 5:**
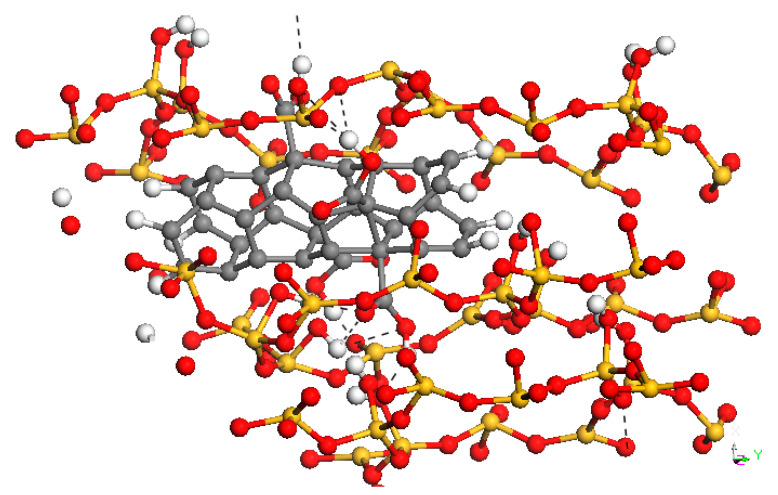
H bonds (black dash lines) between the tobermorite and a CNT functionalized with 4 COOH groups. Red: oxygen, Yellow: silicon, White: hydrogen, Grey: carbon.

As an example, Figure 5 shows the H bonds, as black dashed lines, which are created between the tobermorite and the CNT functionalized with four COOH groups.

According to our above results, the larger the functionalization degree, the better the ISS and E values. The values in Table 5 and Table 6 show that a larger number of carboxyl groups allows for more H bonds at the interface and, consequently, a more favorable non-bond energy. 

## 4. Conclusions

After carrying out MD simulations to study the mechanical properties of a tobermorite matrix, with either a pristine or a functionalized CNT with different concentrations of carboxyl groups, the following conclusions can be highlighted:

- Young’s modulus of tobermorite is enhanced when incorporating nanotubes in its composition;
- Young’s modulus presents higher values when the concentration of carboxyl groups in the CNT is increased. This means that compounds with functionalized nanotubes show better mechanical properties, if compared to pristine nanotubes;
- The obtained values for E are significantly higher than those obtained by other authors with experimental techniques, since it is not possible to simulate the porosity of the cement matrix. When correcting the values with the equation proposed by Knudsen and Helmuth [66], it is possible to obtain values with an order of magnitude very similar to other authors, keeping the tendency of a higher E as a function of the number of functional groups; and
- The functionalization of CNT with carboxyl groups promotes the formation of a H-bond network with tobermorite. The larger the number of functional groups, the more H bonds established at the interphase, causing enhanced adhesion and thus, improving mechanical properties.

## Figures and Tables

**Table 1:** Models used to calculate mechanical properties.

Mechanical Property	Lattice Parameter (Å)	SWCNT	SWCNT Length (Å)
Young’s Modulus (E) Poisson’s Ratio (ν) Shear Modulus (G) Bulk Modulus (K)	a = 20.205 b = 36.920 c = 44.974	Pristine 2 COOH 4 COOH	10.16
Interfacial Shear Strength (τ)	a = 500 b = 29.540 c = 44.974	Pristine 4 COOH 6 COOH	17.24

**Table 2:** Mechanical properties.

Composition	E (GPa)	ν	K (GPa)	G (GPa)
T	57.98	0.26	40.24	23.03
T + CNT	60.56	0.22	36.52	24.74
T + CNT + 2COOH	76.74	0.27	55.18	30.13
T + CNT + 4COOH	83.03	0.29	65.79	32.21

**Table 3:** Young’s modulus and porosity.

Composition	E (GPa)	E (GPa) *p* = 0.26	E (GPa) *p* = 0.36
T	57.98	23.95	17.05
T + CNT	60.56	25.02	17.81
T + CNT + 2COOH	76.74	31.70	22.56
T + CNT + 4COOH	83.08	34.33	24.43

**Table 4:** ISS and non-bond energy.

System	ISS (MPa)	ΔE_non-bond_ (kJ/mol)
Tobermorite + pristine CNT	220.99	18744.32
Tobermorite + CNT 4-COOH	280.85	23819.51
Tobermorite + CNT 6-COOH	491.07	41651.72

**Table 5:** Number of H bonds and their average length (E models).

Structure	NHb	Distance Hb (Å)
T + CNT	0	1.95 *
T + CNT + 2 COOH	2.6	1.83
T + CNT + 4 COOH	9.6	1.82

* close contact.

**Table 6:** Number of H bonds and their average length (pull-out models).

Structure	NHb	D (Å)
Tobermorite + pristine CNT	0	2.06 *
Tobermorite + CNT 4-COOH	6	1.81
Tobermorite + CNT 6-COOH	10	1.79

* close contact.

## Data Availability

Not applicable.
